# Impact of Cold Plasma Treatment on the Shelf Life and Metabolite Profiles of Strawberries during Storage

**DOI:** 10.1021/acsfoodscitech.5c00700

**Published:** 2025-10-03

**Authors:** D. Abouelenein, G.G. Gebremical, S. Tappi, B. Cellini, F. Shanbeh Zadeh, A.M. Mustafa, G. Caprioli, Nadezhda Frolova, A. Soboleva, A. Frolov, L. Vannini, P. Rocculi, S. Vittori

**Affiliations:** † School of Pharmacy, Chemistry Interdisciplinary Project (CHIP) via Madonna Delle Carceri, University of Camerino Camerino 62032, Italy; ‡ Research Group for Analytical Food Chemistry, National Food Institute, Technical University of Denmark (DTU), Kemitorvet 202, Lyngby dk-2800, Denmark; § Department of Agricultural and Food Sciences, University of Bologna, Piazza Goidanich, 60, Cesena, FC 47521, Italy; ∥ Interdepartmental Centre for Agri-Food Industrial Research, University of Bologna, Via Q. Bucci 336, Cesena, FC 47521, Italy; ⊥ Department of Pharmacognosy, Faculty of Pharmacy, Zagazig University, Zagazig 44519, Egypt; # Laboratory of Analytical Biochemistry and Biotechnology, 28403 K.A. Timiryazev Institute of Plant Physiology of Russian Academy of Science, Moscow, Idaho 127276, Russia; ∇ Leibniz Institute of Plant Biochemistry, Department of Bioorganic Chemistry, Halle (saale) 06120, Germany

**Keywords:** nonthermal processing, microbial inactivation, primary metabolites, secondary metabolites, ascorbic acid, quality

## Abstract

Strawberries are abundant in bioactive compounds and serve as a significant source of ascorbic acid. However, their shelf life is notoriously short due to their high sensitivity to environmental conditions and susceptibility to microbial contamination. The growing demand for ready-to-eat products among consumers presents challenges related to food preservation, especially with increasing food losses due to microbial spoilage. Various strategies have been explored to address these issues, one of which is atmospheric cold plasma. A surface dielectric barrier discharge cold plasma (CP) treatment was applied to fresh strawberries (output voltage: 6 kV, frequency: 23 kHz) for 30 min. Spoilage microbial populations, quality, and primary and secondary metabolite profiles were evaluated during storage at 4 °C. Cold plasma treatment resulted in significant reductions in the counts of the main spoilage microbial groups, which showed delayed and limited growth compared to the untreated fruits. LC–MS/MS analysis revealed the preservation of the total phenolic profile, along with a significant increase in total phenolic acids (from 614.2 to 784.4 mg/kg) and total flavonols (from 145.2 to 196.4 mg/kg) immediately after treatment. An increase in the levels of specific polyphenols and antioxidant activity was observed. Although ascorbic acid decreased after treatment, greater stability was noted during storage. In conclusion, CP can preserve fruit quality and extend the shelf life of fresh strawberries without adversely affecting their physical properties.

## Graphical abstract

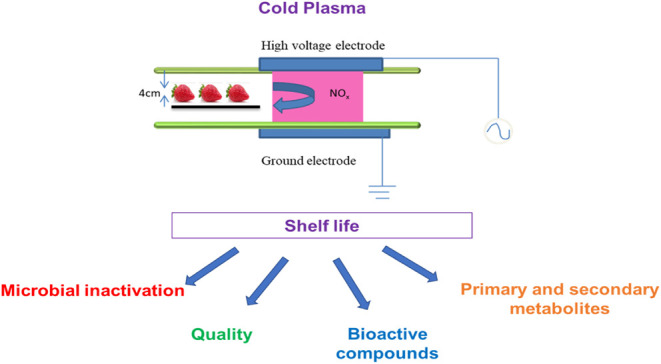

## 1 Introduction

Strawberry (*Fragaria x ananassa*) is one of the most widely spread planted fruit crops worldwide, representing a good source of vitamin C and a wide array of bioactive compounds such as polyphenols, flavonoids, anthocyanins, and tannins^1,2^ and excellent organoleptic properties.^3^ Strawberries have a short postharvest shelf life, as their fruits are susceptible to mechanical injury, physiological deterioration, and microbial spoilage during storage.^4^ Therefore, their preservation is a challenging task. Consequently, improving the shelf life of strawberry fruits by all means including postharvest treatments is absolutely mandatory. The food processing industry faces challenges in preserving fruits while maintaining their quality and safety and extending their shelf life.^5^ Traditional methods like chlorine-based compound washing are commonly used but have drawbacks such as high energy consumption, chemical use, and wastewater production, leading to environmental and human health concerns;^2,6^ therefore, their use is strictly regulated.^7^ For example, fruit decontamination by chlorine-based washing, as well as washing fresh and fresh-cut products is currently prohibited in many countries.^8^ The evaluation of alternative nonthermal technologies is essential for extending the shelf life of food products in general and strawberries specifically without affecting their nutritional value.

In this context, being a novel dry and nonthermal chemical-free technology, atmospheric pressure cold plasma (CP) offers distinct advantages for food decontamination and processing. The term “plasma” is the fourth state of matter and refers to an electrically neutral gas composed of molecules, atoms, ions, and free electrons, evolved using a gas at atmospheric pressure, e.g., ambient air with no need for pumps or vacuum chambers.^9^ Several approaches can be employed for plasma generation: corona discharge, glow discharge, dielectric barrier discharge (DBD), high-frequency discharge, and microwave discharge.^9,10^ The numerous processing parameters that can be modulated allow for large adaptability of the process to the intended use;^11^ however, they also represent high variability that makes difficult the comparison of the obtained results.

Moreover, the highly reactive species present in the plasma discharge have raised concerns related to the formation of potentially toxic byproducts, particularly in high-lipid matrices. However, despite the limitations that this technology still presents, CP has shown very promising results in the food sector generally, and in particular for fruit and vegetables products,^12^ and is therefore worthy of further investigation.

Previous studies have reported the positive effects of CP on decontamination of both fresh^13–15^ and fresh-cut strawberries^16^ and strawberry juice.^17^ While such studies have shown the good potential of CP treatments for reducing microbial loads, only total viable counts and yeasts/molds have been addressed. To date, studies on the efficacy of CP on other microbial populations relevant to the spoilage of strawberry fruit are still scarce. Moreover, previous studies have focused on the immediate effects of the treatments, with less research focusing on the fate of the surviving microbiota during refrigerated storage, which overall is responsible for the fruit’s shelf life. In addition, the effects of CP on the nutritional quality of whole fresh strawberry fruits have only minimally been addressed so far.

This fruit is rich in natural antioxidants and other bioactive compounds and is recognized therefore for its pronounced anticancer, antioxidative, and anti-inflammatory properties.^18^ The nutritional value of strawberry fruits is mainly attributed to the high contents of multiple natural products, mostly represented by phenolics. The importance of dietary phenolics is to a large extent attributed to their high antioxidant activity.^18,19^ The most frequently identified classes of phenolics in strawberries are represented by anthocyanins (which are responsible for the bright red color), phenolic acids, and hydrolyzable tannins. High contents of flavan-3-ols, epicatechin, and procyanidin derivatives have been reported for this fruit species.^18^ Further phenolics present in strawberries in lower concentrations include flavonols, which are dominated by glycosides of quercetin and kaempferol. However, the presence of procyanidins in fruits results in an astringent and unfavorable taste.^20^

The organoleptic properties of strawberries depend mainly on the presence of a broad range of primary metabolites, amino acids, sugars, and organic acids. Sugars define the sweetness of the fruits, while organic acids influence their acidity. Importantly, these metabolites not only affect the quality of strawberries but also play an important role in the development and maturation of their fruits.^19^

Numerous studies have delved into the impact of CP treatments on parameters such as shelf life, food safety, and microbiological changes.^5^ It has been shown to influence the presence of bioactive compounds, including phenolic compounds,^21,22^ ascorbic acid, and antioxidant activity^5^ in various fruit and vegetable matrices. However, with regard to the effect on bioactive compounds, the research on fruit metabolites has been mainly limited to the phenolic profiles.^11,23^ Therefore, the objective of this study is a comprehensive investigation, offering valuable insights to enhance our understanding of the effects of CP on microbial decontamination and the quality of strawberries, including primary and secondary metabolites during storage.

To achieve this, HPLC–MS/MS was utilized to simultaneously quantify 38 bioactive phenolic compounds in strawberries during refrigerated storage. Additionally, the study addressed the effects of CP treatment on the total contents of polyphenols and flavonoids, as well as the associated antioxidant activity. An untargeted GC–MS analysis of primary metabolites was also conducted and comprehensively analyzed using both multivariate and univariate statistical methods.

## 2 Materials and Methods

### 2.1 Samples

Fresh strawberries (*Fragaria × ananassa*), locally grown and harvested in July 2023, were purchased at the local market in Cesena, Italy and stored in a refrigerated chamber at 2 ± 1 °C for a maximum of 24 h before treatment. The selected strawberries had a consistent physiological and maturity state without any visible damage. Fruits were selected randomly to be treated with CP (named T) or used as the control group (named C).

### 2.2 Cold Plasma Treatment

CP was generated by a surface dielectric barrier discharge (SDBD) placed at the top of a closed chamber, defining a confined atmosphere. A high-voltage generator produced a sinusoidal waveform with a peak voltage of 6 kV, a frequency of 23 kHz, a power density of 425.35 ± 25.79 W, and a surface power density of 2.6 W cm^–2^. The major reactive species formed with the same generator and operating conditions are NO_*x*_, as reported in previous research.^24^ The distance measured from the surface of the SDBD to the top surface of the fruit sample was 10 cm. Treatments were carried out at room temperature (26 ± 1 °C) for 30 min. The treatment duration was selected in previous experiments as the time necessary to reach a significant (*p* < 0.05) inactivation of approximately 1–2 Log CFU g^–1^ for the target spoilage populations. Each treatment was carried out in 3 independent replicates with 36 fruits each.

### 2.3 Storage

After treatment, samples were packed in plastic macroperforated trays containing 9 fruits each and subjected to storage at 4 °C for up to 6 days. After 0, 1, 3, and 6 days, 3 trays from the control group (C) and 3 trays from the plasma group (T) were selected for analytical determinations. Microbiological analysis and quality determinations were carried out on the fresh products as specified below, while all the remaining fruit samples were frozen at −80 °C, freeze-dried, and then subjected to chemical analysis on the dried mixture.

### 2.4 Microbiological Analysis

A 10 g portion, prepared using exclusively the external part (approximately 1–1.5 mm) of 4/5 fruits, was serially diluted in sterile saline solution (0.9% NaCl) and homogenized in a Stomacher (120 s). Dilutions were then plated on different media to determine the cell counts of different spoilage microbial populations. Total mesophilic and psychrotrophic bacteria were enumerated on Plate Count Agar (PCA; Oxoid, Milan, Italy), Enterobacteriaceae on Violet Red Bile Glucose Agar (VRBGA; Oxoid, Milan, Italy), yeasts and molds on Yeast Peptone Dextrose (YPD; Oxoid, Milan, Italy) supplemented with chloramphenicol (200 mg L^–1^), lactococci and lactobacilli on M17 (Oxoid, Milan, Italy) and De Man-Rogosa-Sharpe Agar (MRS; Oxoid, Milan, Italy) supplemented with cycloheximide (200 mg L^–1^), respectively. Plates were incubated for 48 h at 30 °C for YPD and PCA (for mesophilic bacteria), 10 days at 8 °C for PCA (for psychrotrophic bacteria), 48 h at 37 °C in anaerobic conditions for MRS and M17, and 24 h at 37 °C for VRBGA. Three independent replicates were conducted for plasma treatment, and results of microbial counts at each time point were expressed as mean Log CFU g^–1^ ± SD.

### 2.5 Determination of Quality Parameters (Color, Hardness, TSS, and Dry Matter)

For quality parameters, 10 fruits from the 3 packages were used for each sampling date and sample. To measure the color of strawberries, a spectrophotocolorimeter (Color Flex, 40/0, HunterLab, Reston, VA, USA) was used. The instrument was calibrated before use and set with a D65 illuminant and the 10-standard observer. The parameters L* (lightness), a* (greenness), and b* (yellowness) were obtained. Ten strawberries per sample were analyzed, and for each strawberry, measurements were obtained from 2 opposite sides (20 measurements).

Texture measurements were conducted by means of a texture analyzer (TA-HDi, Stable Micro Systems, UK) equipped with a 5 kg load cell. To determine the texture of strawberries, each fruit was cut in half, and a penetration test was applied using a 2 mm cylinder probe to puncture them in the center. The probe moved with a cross-head speed of 1 mms^–1^ and a trigger force of 1 g until a maximum deformation of 90%. The pretest and post-test speeds were set at 10 mm s^–1^. The acquired curves (force (N) vs time (s)) were analyzed, and the hardness (the maximum peak force) value (N) was extracted.

Total soluble solids (TSSs) were determined using a digital refractometer (Model: DPR 95), and dry matter content was determined by drying the sample in an oven at 70 °C until a constant weight was achieved.

### 2.6 Ascorbic Acid Content

The content of ascorbic acid was determined by an HPLC-DAD method previously described by ref 25, in which 20 mg of freeze-dried samples were immersed in a 1 mL extraction solution of water containing 1% meta-phosphoric acid aqueous solution. The extraction was performed for 1 h in darkness using a magnetic stirrer. Then, the samples were centrifuged at 15,000*g* for 10 min. The clear supernatant was filtered using a 0.45 μm membrane filter before analysis.

### 2.7 Determination of DPPH Radical Scavenging Activity

The free-radical scavenging capacity in strawberry samples was measured spectrophotometrically at a wavelength of 517 nm, using the stable radical 2,2-diphenyl-1-picrylhydrazyl (DPPH^•^) according to the method used by ref^18^. Trolox (6-hydroxy-2,5,7,8-tetramethylchroman-2-carboxylic acid) was used as a reference standard. Results were expressed in milligrams of Trolox equivalent g^–1^ of dry matter.

### 2.8 Determination of Total Phenolic Content

The total phenolic content of the strawberry samples was measured spectrophotometrically using an Agilent Cary 8454 UV–Vis spectrophotometer at 765 nm, through the Folin–Ciocalteu method described by ref 25. Gallic acid was used as the polyphenolic reference standard, and the results were expressed in milligrams of gallic acid equivalent g^–1^ of dry matter.

### 2.9 Analysis of Phenolic Profile by LC–MS/MS

30 mg of freeze-dried samples were extracted with 1.5 mL of methanol/formic acid (0.1% v/v). The samples were homogenized using a vortex for 1 min and then sonicated (FALC ultrasonic bath, Treviglio, Italy) at a frequency of 40 kHz for 30 min at 25 °C. Subsequently, samples were centrifuged at 15,000*g* for 10 min with a Thermo Scientific IEC CL10 Centrifuge from Thermo Electron Industries SAS (Château-Gontier, France). The resultant supernatant was filtered through a 0.2 μm syringeless filter before injection into the HPLC–MS/MS triple quadrupole. Extractions were performed and analyzed in triplicate.

Phenolic identification and quantification were completed as previously reported by ref 18. Identification was carried out on an Agilent 1290 Infinity series and a Triple Quadrupole 6420 from Agilent Technologies (Santa Clara, CA), equipped with an electrospray ionization (ESI) source operating in negative and positive ionization modes. The chromatographic separation was achieved using a Synergi Polar-RP C18 analytical column (250 mm × 4.6 mm, 4 μm) from Phenomenex (Cheshire, UK). The elution method used was a binary gradient, A [water/formic acid, 0.1% v/v] and B [methanol/formic acid, 0.1% v/v], applied in the following gradients: 0–1 min, isocratic condition, 20% B; 1–25 min, 20–85% B; 25–26 min, isocratic condition, 85% B; 26–32 min, 85–20% B. The flow rate was 0.8 mL min^–1^, the injection volume was 2 μL, and the column temperature was set at 30 °C. The temperature of the drying gas in the ionization source was 350 °C. The gas flow was 12 L min^–1^, the nebulizer pressure was 55 psi, and the capillary voltage was set at 4 kV. Detection was performed in the dynamic multiple reaction monitoring (dynamic- MRM) mode, and the dynamic-MRM peak areas were integrated for quantification. The most abundant product ion was used for quantitation, and the others were used for qualification. The specific time window for each compound (Δ retention time) was set at 2 min. The selected ion transitions and the mass spectrometer parameters for the analyzed compounds are reported in Table S1.

### 2.10 GC–MS Analysis of Silylated Primary Metabolites

#### 2.10.1 Metabolome Extraction and Two-Step Derivatization

Metabolites were extracted from strawberry samples according to the method reported by ref 26, with some modifications. The extraction solution was prepared by dissolving adonitol (internal standard; IS) into methanol to obtain a 50 μmol mL^–1^ solution. Freeze-dried samples (20 mg each) were suspended in 800 μL of extraction solvent, vortex-mixed, and then incubated in an ultrasonic bath for 10 min, followed by centrifugation at 15,000*g* for 10 min. The supernatant was collected, and the extraction was repeated with 400 μL of water to obtain a total of 1100 μL of extract. 300 μL of hexane was added to remove unwanted lipids, and then samples were vortexed shortly and centrifuged for 5 min at 2,000*g*. The lower layer containing the metabolites was transferred into a new microcentrifuge tube. Pooled extracts were prepared to be used as quality controls (QC) by mixing equal aliquots of samples. Process/extraction blanks were also prepared by following the same extraction procedure described above without including the samples.

For derivatization, 20 μL of each metabolomic extract was evaporated using a vacuum evaporator (Eppendorf Concentrator Vacufuge Plus, AG, Germany No. 022820168). In the first step, 30 μL of methoxamine (MEOX) hydrochloride (20 mg mL^–1^ in pyridine) was added to each sample, then incubated at 30 °C for 90 min under shaking. In the second step, metabolites were converted into trimethylsilyl (TMS) derivatives using 55 μL of MSTFA (*N*-trimethylsilyl-*N*-methyl trifluoroacetamide), and then samples were shaken for 30 min at 37 °C at a speed of 750 rpm, according to the earlier established procedure.^27^ Finally, a mixture containing *n*-alkanes (C8 to C40, 5 μg mL^–1^) was added to the vials and injected to further calculate retention indices (RI) and to evaluate instrument performance.

#### 2.10.2 Metabolomic Analysis

Derivatized samples were analyzed by gas chromatography–electron ionization-quadrupole-mass spectrometry (GC–EI-Q-MS) using a GC2010 gas chromatograph coupled online to a quadrupole mass-selective detector, Shimadzu GCMS QP2010, equipped with a CTC GC PAL liquid injector (Shimadzu Deutschland GmbH, Duisburg, Germany). Samples were randomly separated and injected together with five quality control (QC) samples, a process/extraction blank, and a derivatization control. The derivatization control is a mixture of the derivatization reagents added following the same derivatization procedure as that of samples, which was used to allow further exclusion of features related to the derivatization reagents. The GC was equipped with an HP-5 capillary column (30 m × 0.25 mm ID, 0.25 μm film thickness, Thermo Fisher Scientific, Bremen, Germany). The oven temperature was held at 40 °C for 1 min, increased to 70 °C at 15 °C min^–1^ and held for 1 min, then increased to 320 °C at 6 °C min^–1^ and held for 10 min. Helium was used as a carrier gas at a rate of 1 mL min^–1^. The MS detector was operated in EI positive mode (scan range of *m*/*z* 50–500, resolution of 60,000). The transfer line and ion source were maintained at 250 °C. Injection (1 μL) was performed in splitless mode (90 s of splitless time). Gas chromatographic (GC) separation conditions and electron ionization-quadrupole-mass spectrometry (EI-Q-MS) settings are reported in Table S2.

#### 2.10.3 GC Data Preprocessing and Metabolomic Identification

The preprocessing steps were performed using the Automated Mass Spectral Deconvolution and Identification System (AMDIS, version 2.66 from 08.08.2008, freely available via www.amdis.net) and Xcalibur (version 2.0.7) in order to perform peak deconvolution and alignment of features from samples.^28^ The preprocessing procedure included importing raw files (mzML data), followed by mass detection and the construction of extracted ion chromatograms to build a separate chromatogram – EIC – for each *m*/*z* detected by the instrument. Then, peak detection and deconvolution were applied to integrate peaks from the EIC and to construct mass spectra of features by combining peaks from different EICs, respectively, thereby resulting in a list of RT-*m*/*z* to generate a table with all features detected. A filtering step was further applied to remove features associated with extraction blanks and derivatization controls.

The mass spectra of all detected compounds were identified by comparison with available spectral libraries – National Institute of Standards and Technology (NIST), Golm Metabolome Database (GMD), Human Metabolome Database (HMDB), and an in-house library (partly with Kovats retention time indices, calculated from the retention times of alkane standards). Quantitation relied on integration of the corresponding extracted ion chromatograms (XICs, ± 0.5 Da) at specific retention times (*t*_R_).

### 2.11 Statistical Analysis

For untargeted primary metabolic analysis, the data matrix was exported using Excel (Excel 365, Microsoft, Redmond, WA, USA) for all samples, including their replicates. The dataset was then modeled, and the processing and statistical interpretation of the acquired data relied on the MetaboAnalyst 5.0 online platform (freely available via www.metaboanalyst.ca). Features were filtered if their RSDs were >30% in quality controls, and samples were Pareto-scaled (mean-centered and divided by the square root of the standard deviation of each variable). Principal component analysis (PCA) was applied for checking the general trend in an unsupervised way. Then, partial least-squares discriminant analysis (PLS-DA) was used to maximize the fitness of variables discriminating between the two groups in a supervised way. The PLS-DA model was tested by cross-validation, and the validated model was further considered in sparse PLS-DA (sPLS-DA). In cross-validation, *R* ^2^ indicates the fitness of the PLS-DA model with the whole dataset, while *Q* ^2^ is an estimate of the predictive ability of the model. High *Q* ^2^ values indicate good prediction.^29^ Based on the high number of features in the untargeted metabolome, sPLS-DA was chosen to select the most predictive or discriminative features in the data that help classify the samples.^29,30^

Hierarchical Clustering Analysis (HCA) was used to process data from the HSPME-GC/MS analysis. For other analysis, a one-way analysis of variance (ANOVA), followed by the Tukey post hoc test was used to analyze statistical significance (*p* < 0.05). The data were expressed as means ± SD. Measurements were made in triplicate.

## 3 Results and Discussion

### 3.1 Microbiological Analysis

To evaluate the efficacy of plasma treatment in improving microbial stability and shelf life of strawberries, microbiological analyses were performed by addressing not only total bacterial counts and yeast/molds but also considering different microbial populations typically associated with fresh berries, which could contribute to their deterioration. Viable counts of total mesophilic and psychrotrophic populations, Enterobacteriaceae, yeasts and molds, lactococci, and lactobacilli were detected immediately after the CP treatment and over 6 days of refrigerated storage, as reported in Figure 1. Not only microbial contamination but also subsequent microbial growth play in fact a crucial role by significantly impacting the strawberry postharvest shelf life, which is generally limited to a few days when stored under ordinary atmospheric conditions The highest contamination values of the untreated fruits were detected for mesophiles, psychrotrophs, and yeasts (4.8–5.1 Log CFU g^–1^), while loads ranging between 3.5 and 4.3 Log CFU g^–1^ were found for molds, enterobacteria, and lactococci. In contrast, contamination levels of lactobacilli were the lowest, being close to 3 Log CFU g^–1^. Under the CP processing conditions, no significant (*p* > 0.05) difference was observed in lactobacilli counts between the treated and control samples, suggesting that lactobacilli were the most resistant population (Figure 1e). On the contrary, significant (*p* < 0.05) changes were detected after the plasma treatment for all other microbial groups, indicating different susceptibilities to the plasma process. Overall, the treatment successfully affected the target spoilage microbiota, achieving reductions of 2 to 3 Log immediately post-treatment. These findings suggest that cold plasma effectively impacted microbial cells, potentially through mechanisms such as cell wall and membrane permeabilization, significant intracellular protein damage, or direct chemical damage to nucleic acids by plasma-generated reactive species,^31,32^ e.g., reactive nitrogen species (RNS)-induced oxidative damage and nucleic acid and protein leakage.^33^ The immediate inactivation values achieved in this study are in accordance with available literature on strawberry fruit, although different plasma generation methods and processing conditions were adopted.^34^ It was observed that the count of total microbiota was 0.90 Log CFU g^–1^ lower in strawberries following a 10 min CP treatment. Such a significant reduction resulted in the retention of the microbial load at lower levels during 15 days of storage at both 1 and 6 °C, compared to the control samples. Similarly, Yang et al.^35^ found that a 10 min indirect plasma-processed air pretreatment effectively reduced surface bacterial and fungal contamination by 2 Log units. Moreover, the surviving populations were consistently decreased in the treated strawberries compared to the control fruits throughout 15 days of storage at 10 °C. Inhibition of microbial growth has also been observed in other fresh and fresh-cut products treated with cold plasma, such as melons,^36^ strawberries (M. Li et al., 2019),^100^ and pitayas.^37^

**Figure 1:**
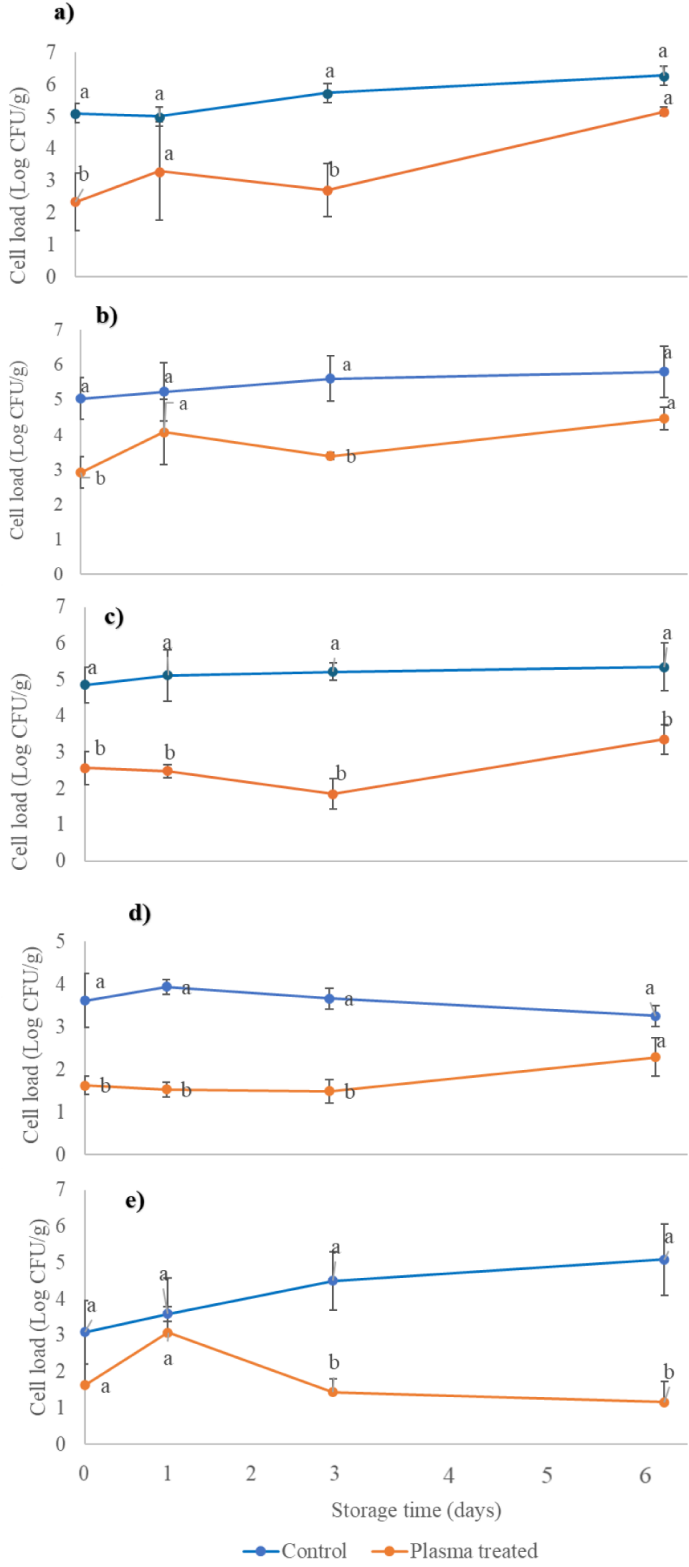
Effect of cold plasma treatment on total mesophilic (a), psychrotrophic bacteria (b), yeasts (c), molds (d), and lactobacilli (e) counts in strawberry fruit during 6 days of storage at 4 °C. Different letters indicate significant differences between control and plasma-treated samples at the same storage time (Tukey HSD, *p* < 0.05).

Likewise, data collected in this study over refrigerated storage showed that the CP treatment affected the fate of the target spoilage microbial populations. While bacterial levels of total mesophilic bacteria were significantly reduced (*p* < 0.05) compared to the untreated control immediately following the treatment, growth of the surviving microbiota was observed over time (Figure 1a). In particular, from the third day onward, bacterial levels gradually increased, reaching final values of up to 5 Log CFU g^–1^ in treated fruits. However, a slower growth rate and a lower final cell count were found for the treated strawberries compared to the control, which exceeded 6 Log units after 3–4 days. Similar dynamics were observed also for the psychrotrophic population, which never exceeded 4.5 Log CFU g^–1^ in the treated samples (Figure 1b). As shown in Figure 1c,d, the shelf-life study of strawberries treated with CP demonstrated an immediate significant (*p* < 0.05) reduction in both total yeast and mold counts, effectively suppressing their growth up to the third day of storage. While counts of both fungal populations gradually increased thereafter, they remained significantly lower (2 and 1 Log CFU g^–1^, respectively) in the cold plasma-treated group than in the control one throughout storage (*p* < 0.05).

The CP treatment reduced also the lactobacilli load and limited their growth to 3.1 Log CFU g^–1^ on the first day, despite no significant differences (*p* > 0.05) being detected compared to the control (Figure 1e). However, over storage, their loads decreased to 1.1–1.4 Log units in the treated samples, suggesting that surviving cells had been strongly injured by the treatment and could not recover from the damage. Likewise, lactococci did not show any growth ability in the treated fruit nor did Enterobacteriaceae, which were randomly detected over storage, while cell viabilities between 3 and 4 Log cycles were recorded for enterobacteria in the control fruits (data not shown).

Overall, despite the immediate cell reductions, growth of the surviving microbiota was observed during storage for some of the selected microbial groups, indicating different sensitivities between fungi and bacteria and between bacterial groups to the plasma treatment. On the other hand, the observed higher susceptibility of fungi to CP treatments compared to mesophilic bacteria is in agreement with findings reported by several authors for strawberries.^14,15^ Mesophilic and psychrotrophic aerobic populations were the primary contributors to spoilage, showing the highest growth potential and exceeding 6 Log CFU g^–1^ within 4–5 days in the control fruits. Despite the large microbial populations present on the strawberry surface, plasma treatment resulted in slower and limited growth for the target spoilage microbiota and increased the shelf life by at least 2–3 days, as the threshold level, i.e., 6 Log units, was not achieved over 1 week.

### 3.2 Physico-Chemical Parameters

#### 3.2.1 Hardness, Dry Matter, TSS, and Color of the Strawberries

Table 1 shows the effects of CP treatment on the hardness, total soluble solids (TSS), dry matter, and color of strawberries. When fresh and fresh-cut produce are exposed to cold plasma treatment, their quality changes depending on the specific parameters of the cold plasma process. Various studies in the literature report differing outcomes based on these conditions.^38^ Regarding our study, the plasma-treated samples exhibited a slight increase in dry matter, while color parameters (L*, a*, and b*) did not differ significantly (*p* > 0.05) between treated and untreated samples, aligning with findings by refs 39, 40 . Additionally, after a 30 min treatment, the hardness of the strawberries was not significantly different from the untreated group (*p* > 0.05). TSS, a key indicator of strawberry nutritional quality representing soluble sugar content,^41^ was also measured. Table 1 shows that TSS levels remained consistent between the control and treated groups from the start to the end of storage, indicating stable TSS content over time (*p* > 0.05), consistent with results from Wu et al.^42^ Thus, CP treatment did not significantly impact strawberry quality.

**Table 1:** Changes in Physico-Chemical Parameters of Untreated (C) and Cold Plasma Processed (T) Strawberries During Storage up to 6 Days at 4 °C (Mean Values ± Standard Deviation)[Table-fn tbl1fn1]

		Storage time (days)			
Quality index	Samples	T_0_	T_1_	T_3_	T_6_
Dry matter (g/100 g)	C	8.95 ± 0.92^c^	12.22 ± 0.72^a^	12.03 ± 1.36^a^	10.48 ± 1.9^b^
	T	9.88 ± 0.65^c^	12.11 ± 0.79^a^	13.76 ± 0.76^a^	12.55 ± 1.81^a^
Soluble Solid Content (g/100g)	C	8.2 ± 0.7^a^	9.10 ± 0.2^a^	8.80 ± 0.20^a^	10.2 ± 0.1^a^
	T	9.3 ± 0.1^a^	9.90 ± 0.2^a^	10.0 ± 0.4^a^	9.6 ± 0.1^a^
Hardness (N)	C	0.34 ± 0.13^a^	0.27 ± 0.10^a^	0.24 ± 0.06^a^	0.32 ± 0.17^a^
	T	0.26 ± 0.07^a^	0.32 ± 0.13^a^	0.18 ± 0.07^a^	0.26 ± 0.09^a^
L*	C	34.28 ± 2.63^a^	31.50 ± 2.51^a^	31.77 ± 3.09^a^	32.99 ± 2.43^a^
	T	33.82 ± 2.56^a^	33.81 ± 3.13^a^	30.44 ± 7.67^a^	32,84 ± 2.08^a^
a*	C	32.66 ± 2.69^a^	32.72 ± 3.22^a^	31.99 ± 3.44^a^	34.19 ± 1.21^a^
	T	32.58 ± 3.54^a^	35.58 ± 3.58^a^	31.55 ± 6.42^a^	32.71 ± 2.11^a^
b*	C	20.22 ± 3.25^a^	19.96 ± 4.51^a^	18.63 ± 4.08^a^	20.87 ± 2.83^a^
	T	19.98 ± 3.80^a^	22.30 ± 5.38^a^	18.64 ± 5.22^a^	19.62 ± 3.83^a^

I Different letters among control and treated samples for the same index during storage indicate significant differences (*p* < 0.05) according to one-way ANOVA followed by Tukey’s comparison test.

#### 3.2.2 The Effect of CP on Ascorbic Acid of Strawberries

As shown in Table 2, CP treatment led to a notable 27.2% reduction in ascorbic acid content in strawberries immediately following the treatment (*T*_0_) compared to that of untreated samples. Literature suggests that both applied voltage and treatment duration significantly affect ascorbic acid levels in strawberries.^43^ The 30 min CP treatment used in our study likely contributed to the ascorbic acid reduction observed. This reduction may result from interactions between plasma reactive species and ascorbic acid.^34,44^ A slight, though not statistically significant (*p* > 0.05), decrease in ascorbic acid in whole strawberries after plasma treatment was reported, while Li et al.^16^ observed similar outcomes with fresh-cut strawberries.

**Table 2:** Changes in the Ascorbic Acid, TPC, and DPPH of Untreated (C) and CP Treated (T) Strawberries, During 6-Day Storage at 4 °C[Table-fn tbl2fn1]

		Storage time (days)			
Quality index	Samples	T_0_	T_1_	T_3_	T_6_
TPC (mg gallic acid equiv/g dry matter)	C	22.98 ± 3.5^bc^	29.29 ± 4.0^a^	26.27 ± 1.3^ab^	25.49 ± 5.1^ab^
	T	22.64 ± 3.9^bc^	21.07 ± 1.8^c^	24.83 ± 2.3^bc^	24.93 ± 2.9^bc^
DPPH (mg trolox equiv/g dry matter)	C	24.55 ± 2.14^b^	30.43 ± 4.1^a^	28.31 ± 3.5^b^	24.10 ± 2.8^b^
	T	25.37 ± 6.46^b^	24.18 ± 2.3^b^	29.07 ± 4.1^a^	29.67 ± 5.3^a^
Ascorbic acid (mg/g dry matter)	C	3.82 ± 0.1^b^	4.16 ± 0.4^bc^	4.36 ± 0.2^bc^	4.06 ± 0.5^bc^
	T	2.78 ± 0.7^c^	4.23 ± 0.3^bc^	5.02 ± 0.7^a^	4.87 ± 0.6^a^

I Different letters among control and treated samples for the same index during storage indicate significant differences (*p* < 0.05) according to one-way ANOVA followed by Tukey’s comparison test.

Interestingly, although the ascorbic acid content was reduced immediately after treatment, its stability during storage was notably improved. As shown in Table 2, plasma-treated strawberries consistently maintained higher ascorbic acid levels than did untreated samples throughout storage. This enhanced retention may be attributed to increased cell membrane permeability induced by plasma reactive species, which can facilitate ascorbic acid extractability, a mechanism suggested by Giannoglou et al. (2021). Similarly, Zhou et al.^45^ observed higher maintenance of ascorbic acid levels in cold plasma-treated blueberries during storage compared to the control, suggesting that the treatment inhibits its degradation. According to Ji et al.,^46^ the production of NO during plasma discharge results in production of ascorbic acid as an endogenous antioxidant response, counterbalancing the oxidation caused by plasma reactive species.

#### 3.2.3 The Effect of CP on TPC and DPPH in Strawberries

As shown in Table 2, CP treatment significantly affected the total phenolic content (TPC) or free-radical scavenging activity (DPPH) in strawberries compared to the control only after 1 day of storage, during which it was lower. For the remaining storage times, the phenolic profile remained stable under these treatment conditions. This finding aligns with the LC–MS/MS results shown in Table 3 Although a slight increase in DPPH-reducing activity was noted immediately after treatment, the change was not statistically significant (*p* > 0.05).

**Table 3:** Effect of CP Treatment on Phenolic Compound Content (mg kg^–1^, Dry Weight Basis) of Strawberries (Control = C; Plasma-Treated = T) at Different Storage Times (0, 1, 3, 6 Days) at 4 °C[Table-fn tbl3fn1]

Storage time (days)	0		1			3			6	
Sample	C	T	C		T	C	T		C	T
Gallic acid	11.90 ± 2.72b	16.60 ± 4.70ab	16.78 ± 2.11ab		12.07 ± 1.09b	15.98 ± 2.54ab	15.04 ± 1.63ab		17.87 ± 2.48a	13.53 ± 5.65ab
Neochlorogenic acid	n.d.	n.d.	n.d.		n.d.	n.d.	n.d.		n.d.	n.d.
Chlorogenic acid	2.75 ± 0.92b	3.76 ± 0.89ab	4.90 ± 1.13a		3.15 ± 0.74b	3.58 ± 1.10ab	3.32 ± 0.68b		2.97 ± 0.80b	3.68 ± 0.94ab
Hydroxy benzoic acid	10.29 ± 2.10b	26.26 ± 2.74a	12.20 ± 3.58b		12.70 ± 4.88b	14.75 ± 3.00b	14.35 ± 4.37b		15.10 ± 3.51b	12.31 ± 2.42b
Caffeic acid	3.84 ± 0.74bcd	5.74 ± 1.54a	3.09 ± 0 .38d		3.31 ± 1.07cd	4.46 ± 1.30abcd	4.81 ± 1.01abcd		5.21 ± 0.39ab	5.03 ± 1.13abc
Vanillic acid	n.d.	n.d.	n.d.		n.d.	n.d.	n.d.		n.d.	n.d.
Syringic acid	n.d.	n.d.	n.d.	n.d.		n.d.	n.d.		n.d.	n.d.
*p*-Coumaric acid	113.84 ± 31.96ab	128.36 ± 23.98a	93.95 ± 13.39ab	83.29 ± 18.77b		103.78 ± 11.09ab	127.20 ± 20.84a		122.55 ± 23.96a	112.17 ± 11.29ab
Ferulic acid	1.33 ± 0.11a	1.44 ± 0.24a	1.21 ± 0.17a	1.34 ± 0.11a		1.20 ± 0.21a	1.29 ± 0.14a		1.23 ± 0.22a	1.36 ± 0.25a
3,5-Dicaffeoylquinic acid	n.d.	n.d.	n.d.	n.d.		n.d.	n.d.		n.d.	n.d.
Ellagic acid	470.26 ± 175.46a	602.29 ± 158.41a	669.24 ± 15.24a	475.08 ± 40.64a		664.18 ± 97.44a	536.30 ± 112.53a		643.25 ± 162.26a	509.48 ± 183.43a
Total phenolic acids	**614.20 ± 185.40b**	**784.44 ± 167.08a**	**801.37 ± 25.74a**	**590.95 ± 29.50b**		**807.93 ± 98.76a**	**702.30 ± 90.46ab**		**808.19 ± 176.51a**	**657.58 ± 180.27ab**
Delphidin 3,5 diglucoside	47.87 ± 13.15a	55.03 ± 13.16a	64.06 ± 9.36a	38.14 ± 3.58a		52.88 ± 12.47a	42.40 ± 9.21a		49.80 ± 12.44a	51.07 ± 19.01a
Delphidin3-galactoside	n.d.	n.d.	n.d.	n.d.		n.d.	n.d.		n.d.	n.d.
Cyanidin-3-glucoside	106.28 ± 22.21bc	90.47 ± 29.40c	163.92 ± 21.54a	97.21 ± 14.38c		149.06 ± 20.70ab		120.42 ± 59.89abc	116.34 ± 24.09abc	132.33 ± 23.19abc
Petunidin-3-glucoside	n.d.	n.d.	n.d.	n.d.		n.d.		n.d.	n.d.	n.d.
Pelargonidin-3-rutinoside	591.21 ± 90.64ab	502.07 ± 159.43b	682.89 ± 45.74a	519.69 ± 22.82ab		631.00 ± 107.73ab		595.44 ± 132.38ab	674.37 ± 96.39a	641.02 ± 39.69ab
Pelargonidin-3-glucoside	4862.94 ± 1050.91ab	4694.33 ± 1391.04b	6599.08 ± 1736.32a	4633.84 ± 678.93b		5743.00 ± 612.39ab		5446.27 ± 1133.82ab	6020.59 ± 946.62ab	5569.89 ± 802.44ab
Malvidin-3-galactoside	n.d.	n.d.	n.d.	n.d.		n.d.		n.d.	n.d.	n.d.
Total anthocyanins	**5608.30 ± 1146.88ab**	**5341.90 ± 1580.82b**	**7509.95 ± 1782.76a**	**5288.89 ± 713.01b**		**6575.94 ± 689.68ab**		**6204.53 ± 1325.12ab**	**6861.11 ± 954.73ab**	**6394.32 ± 880.45ab**
Phloridzin	65.82 ± 12.52a	67.06 ± 17.91a	80.76 ± 18.13a	59.81 ± 12.39a		73.72 ± 15.84a		78.79 ± 21.07a	67.29 ± 13.42a	77.51 ± 10.16a
Phloretin	0.12 ± 0.02ab	0.10 ± 0.03ab	0.14 ± 0.03a	0.10 ± 0.02ab		0.11 ± 0.01ab		0.11 ± 0.03ab	0.09 ± 0.01b	0.08 ± 0.01b
Total dihydro chalcones	**65.93 ± 12.54a**	**67.16 ± 17.93a**	**80.89 ± 18.17a**	**59.91 ± 12.38a**		**73.83 ± 15.84a**		**78.90 ± 21.09a**	**67.38 ± 13.42a**	**77.59 ± 10.16a**
Catechin	1030.12 ± 244.08b	987.53 ± 247.95b	1634.49 ± 351.49a	950.05 ± 167.78b		1323.98 ± 180.57ab		1336.76 ± 194.92ab	1095.54 ± 289.68b	1355.80 ± 167.19ab
Epicatechin	8.97 ± 2.15ab	7.87 ± 1.637ab	11.30 ± 3.38a	7.00 ± 1.55b		10.77 ± 2.24ab		10.42 ± 2.75ab	9.69 ± 1.32ab	11.14 ± 1.60a
Procyanidin A2	n.d.	n.d.	n.d.	n.d.		n.d.		n.d.	n.d.	n.d.
procyanidin B2	136.33 ± 14.28bc	125.44 ± 26.37bc	239.90 ± 55.19a	97.67 ± 26.96c		167.50 ± 22.51b		141.16 ± 22.62bc	149.26 ± 35.14bc	144.91 ± 15.34bc
Total Flavan-3-ols	**1175.42 ± 259.11b**	**1120.84 ± 274.8b**	**1885.69 ± 404.08a**	**1054.72 ± 196.12b**		**1502.24 ± 195.72ab**		**1488.34 ± 217.67ab**	**1254.49 ± 320.10b**	**1511.85 ± 180.45ab**
Quercetin-3-*O*-rutinoside	2.39 ± 0.82c	3.29 ± 0.51ab	2.51 ± 0.59bc	2.45 ± 0.24bc		2.55 ± 0.30bc		2.79 ± 1.24bc	3.59 ± 0.73ab	4.04 ± 0.44a
Quercetin-3-*O*-glucoside	7.07 ± 1.50cd	9.78 ± 1.37ab	9.23 ± 0.88abcd	6.56 ± 1.01d		9.56 ± 0.97abcd		8.37 ± 2.20bcd	10.65 ± 1.01b	11.74 ± 3.84a
Quercetin-3-*O*-rhamnoside	n.d.	n.d.	n.d.	n.d.		n.d.		n.d.	n.d.	n.d.
Myricetin	n.d.	n.d.	n.d.	n.d.		n.d.		n.d.	n.d.	n.d.
Kaempferol-3-glucoside	126.23 ± 36.9b	169.76 ± 54.53a	217.97 ± 65.47a	121.27 ± 45.49b		231.34 ± 68.22a		152.33 ± 36.27ab	188.44 ± 36.01a	172.96 ± 50.50a
Quercetin	0.65 ± 0.15bc	0.74 ± 0.01abc	1.00 ± 0.24a	0.62 ± 0.02c		0.94 ± 0.13ab		0.74 ± 0.14abc	0.88 ± 0.25abc	0.96 ± 0.28ab
Isorhamnetin	0.13 ± 0.04ab	0.10 ± 0.02ab	0.14 ± 0.04a	0.09 ± 0.01ab		0.14 ± 0.04ab		0.09 ± 0.01ab	0.12 ± 0.03ab	0.09 ± 0.02b
Quercetin 3-*O*-galactoside	5.67 ± 1.17c	7.98 ± 1.54ab	7.10 ± 0.57bc	5.42 ± 1.25c		8.58 ± 1.26a		7.13 ± 1.66bc	8.85 ± 0.96a	9.97 ± 2.81a
Kaempferol	3.05 ± 0.61b	4.70 ± 1.24a	3.59 ± 1.07b	3.51 ± 0.30b		5.54 ± 1.71a		3.53 ± 0.54b	4.16 ± 0.72ab	3.43 ± 0.96b
Total Flavonols	**145.19 ± 40.16b**	**196.36 ± 53.11a**	**241.54 ± 66.99a**	**139.93 ± 46.80b**		**258.65 ± 69.78a**		**174.98 ± 37.28a**	**216.69 ± 38.56a**	**203.20 ± 57.86a**
Naringin	n.d.	n.d.	n.d.	n.d.		n.d.		n.d.	n.d.	n.d.
Hesperidin	2.10 ± 0.31bc	2.28 ± 0.06abc	2.37 ± 0.62abc	2.18 ± 0.54bc		2.08 ± 0.30c		2.10 ± 0.28bc	2.94 ± 0.68ab	3.11 ± 0.72a
Total Flavanone	**2.10 ± 0.31bc**	**2.28 ± 0.06abc**	**2.37 ± 0.62abc**	**2.18 ± 0.54bc**		**2.08 ± 0.30c**		**2.10 ± 0.28bc**	**2.94 ± 0.68ab**	**3.11 ± 0.72a**
*tran*s-Cinnamic acid	104.90 ± 47.63a	110.07 ± 63.49a	56.78 ± 22.26a	64.99 ± 32.24a		82.61 ± 0.89a		66.67 ± 8.63a	93.21 ± 13.49a	94.52 ± 3.84a
Total phenolics	**7716.0 ± 1620.0b**	**7623.1 ± 1949.3b**	**10578.6 ± 2225.6a**	**7201.6 ± 850.7b**		**9303.3 ± 492.2ab**		**8717.8 ± 1612.1ab**	**9304.0 ± 1439.4**	**8942.2 ± 1220.9ab**

I Different letters in the same row indicate significant differences (*p* < 0.05) according to one-way ANOVA followed by Tukey’s comparison test. n.d.: not detected.

Throughout storage, TPC remained relatively unchanged, while DPPH activity showed a significant increase over time in the treated samples. This boost in antioxidant activity could be associated with the increase in ascorbic acid and specific phenolic compounds observed (Table 2). A similar result was observed by Ji et al.^46^ in blueberries during storage. In contrast, conventional thermal treatments such as blanching, pasteurization, and sterilization often cause polyphenol oxidation and degradation in strawberries. For instance, Garzoli et al.^47^ reported a 46% reduction in total phenolic content and a 64% decline in antioxidant activity due to pasteurization. Enzymatic and mechanical treatments also impact polyphenol levels, underscoring the need for alternative methods that better preserve polyphenols and antioxidant capacity in strawberries.^48^

#### 3.2.4 Phenolic Profile in Strawberries Based on LC–MS/MS

A variety of phenolic compounds were identified in the strawberry samples, totaling 25 distinct compounds, including 7 phenolic acids, 7 flavonols, 4 anthocyanins, 3 flavan-3-ols, 2 dihydrochalcones, and 1 flavanone, alongside *trans*-cinnamic acid. Table 3 presents these phenolic compounds for both control- and plasma-treated strawberry samples across different storage time points. Notably, anthocyanins were the most abundant phenolic group, followed by flavan-3-ols. These phenolic profiles closely matched those reported in the literature.^18^

As illustrated in Table 3, LC–MS/MS analysis showed that the phenolic profile remained stable immediately after plasma treatment, with no significant changes in total phenolic content at time zero (*T*_0_, *p* > 0.05). However, total phenolic acids significantly increased from 614.2 to 784.4 mg kg^–1^, along with flavonols from 145.2 to 196.4 mg kg^–1^. Specifically, *p*-hydroxybenzoic acid increased by 155%, and caffeic acid increased by 49%. Similar increases in phenolic acids, such as chlorogenic acid and caffeic acid, have been observed in plasma-treated samples in previous studies.^17,25^ These increases may be due to the enhanced biosynthesis of *p*-coumaric acid, catalyzed by enzymes like phenylalanine ammonia-lyase (PAL) and cinnamate 4-hydroxylase (C4H) following plasma exposure.^49^ However, this should be confirmed by further analysis.

Flavonol content also rose significantly on day 0, particularly for glucosides such as quercetin-3-*O*-rutinoside, quercetin-3-*O*-glucoside, quercetin-3-*O*-galactoside, and kaempferol-3-glucoside. This trend is consistent with findings in blueberries^45^ and chokeberry juice.^50^ The O-3 sugar group enhances flavonol stability by preventing plasma reactive species oxidation through inhibition of tautomerization between *o*-quinone and *p*-quinonoid forms.^49^ Consistent with previous findings, cold plasma treatment also increased the concentration of quercetin.^49^ Contrary to previous studies suggesting that plasma treatment degrades kaempferol, our study showed a significant increase in kaempferol levels in strawberry post-treatment, rising from 3.1 to 4.7 mg kg^–1^. Generally, phenolic accumulation is a stress response to plasma reactive species generated during plasma treatment.^16^

Anthocyanins, responsible for strawberries’ red color, remained unaffected immediately after processing, with no significant differences between treated and control samples (*p* > 0.05). Given their sensitivity, anthocyanins are typically reduced by traditional processing methods.^51^

With regard to the storage effect, total phenolic content peaked on day 1 and then gradually declined by day 6, with no significant difference compared to the control at time zero (*p* > 0.05). Although a decrease was observed compared to the control, CP-treated strawberries effectively maintained their phenolic content throughout storage. For individual phenolic compounds, treated strawberries largely retained their levels, although specific compounds showed significant (*p* < 0.05) decreases when compared to controls at each storage point. After 1 day, reductions were noted in anthocyanins (pelargonidin-3-glucoside, cyanidin-3-glucoside), flavonols (kaempferol-3-glucoside, quercetin), flavan-3-ols (catechin, epicatechin), and chlorogenic acid. Kaempferol content also showed a significant decline by day 3 in treated samples, though these changes were not significant (*p* > 0.05) compared to controls at day 0. By day 6, quercetin glycosides (quercetin-3-*O*-rutinoside, quercetin-3-*O*-glucoside, and quercetin-3-*O*-galactoside) increased significantly in both treated and control samples. Similar storage-related trends in quercetin and kaempferol derivatives have been reported in strawberries.^52^ Interestingly, a significant increase in hesperidin was noted on day 6 in processed samples only, likely due to polyphenol oxidase (PPO) inactivation resulting from nonthermal plasma treatment.^43^

#### 3.2.5 GC–MS-Based Profiling of Primary Metabolites

Changes in the qualitative and quantitative profiles of primary metabolites, particularly sugars and acids, serve as key indicators of strawberry flavor, taste, and overall quality.^53^ The primary metabolic response to food processing or treatment is complex and varies significantly depending on treatment duration, type, stress level, as well as food matrix characteristics, species origin, and other factors.^53^ Traditional processing techniques such as high-temperature, enzymatic, or mechanical treatments often degrade or destroy sugars and amino acids in strawberries.^54^ Thus, selecting appropriate processing methods and optimizing treatment conditions are essential to minimize the degradation of sugars, acids, and other metabolites, preserving both the sweetness and nutritional value.

Research on the effects of nonthermal plasma on the primary metabolomic profile of fruits and vegetables remains limited, and further studies are necessary to fully understand cold plasma’s impact on primary metabolites and to optimize treatment for health benefits.^48^

Using a GC–EI-Q-MS approach, a total of 122 thermally stable primary metabolites were identified in control and CP-treated strawberry samples as methyl oxime trimethylsilyl (MO-TMS) derivatives. Of these, 107 were identified as primary metabolites through spectral matching or coelution with standards, with 104 unique compounds. These metabolites included 7 amino acids, 29 acids and esters, 21 sugars, and 9 other compounds. Figure S1 shows a representative total ion chromatogram (TIC) of the control strawberry sample, showing the major detected primary metabolites. Additionally, 8 metabolites were partially classified by specific signals (*m*/*z* values), suggesting categories such as cinnamic acids, carboxylic acids, amino acids, and alcohol derivatives. An additional 27 unidentified metabolites were labeled as “Unknown,” with annotations including retention time (tR) and retention index (RI) (Table S3). Monosaccharides, particularly glucose and fructose, were the most abundant, aligning well with earlier studies.^37,55^

When comparing metabolite levels before (C_0_) and immediately after CP treatment (T_0_), no clear separation between control and treated samples was observed using PCA (Figure S2A). Similarly, the PLS-DA model (Figure S2B) showed poor performance, with a negative *Q* ^2^ score from cross-validation, indicating that the model was not predictive and may have been overfitted.^56^ This poor performance suggests that any differences in metabolite profiles between control and plasma-treated samples were minimal or undetectable with the current sample size (*n* = 3). The lack of clear differentiation between the control and SDBD-treated samples suggests that there were no significant differences in the metabolite profiles immediately after treatment.

To compare the changes in the relative metabolite contents observed before and after CP treatment, samples from different storage days, i.e., before (C_0_ to C_6_) and after treatment (T_0_ to T_6_), were analyzed. For better feature selection and data reduction, supervised partial least-squares discriminant analysis (PLS-DA) and sparse partial least-squares discriminant analysis (sPLS-DA) were applied and statistically analyzed. A trend of separation in the metabolome profiles based solely on the storage period was observed, where a clear separation in the sPLS-DA between samples treated on day 6 were grouped on the left side of the scores plot and almost all other samples clustered separately in the right sector (Figure 2).

**Figure 2:**
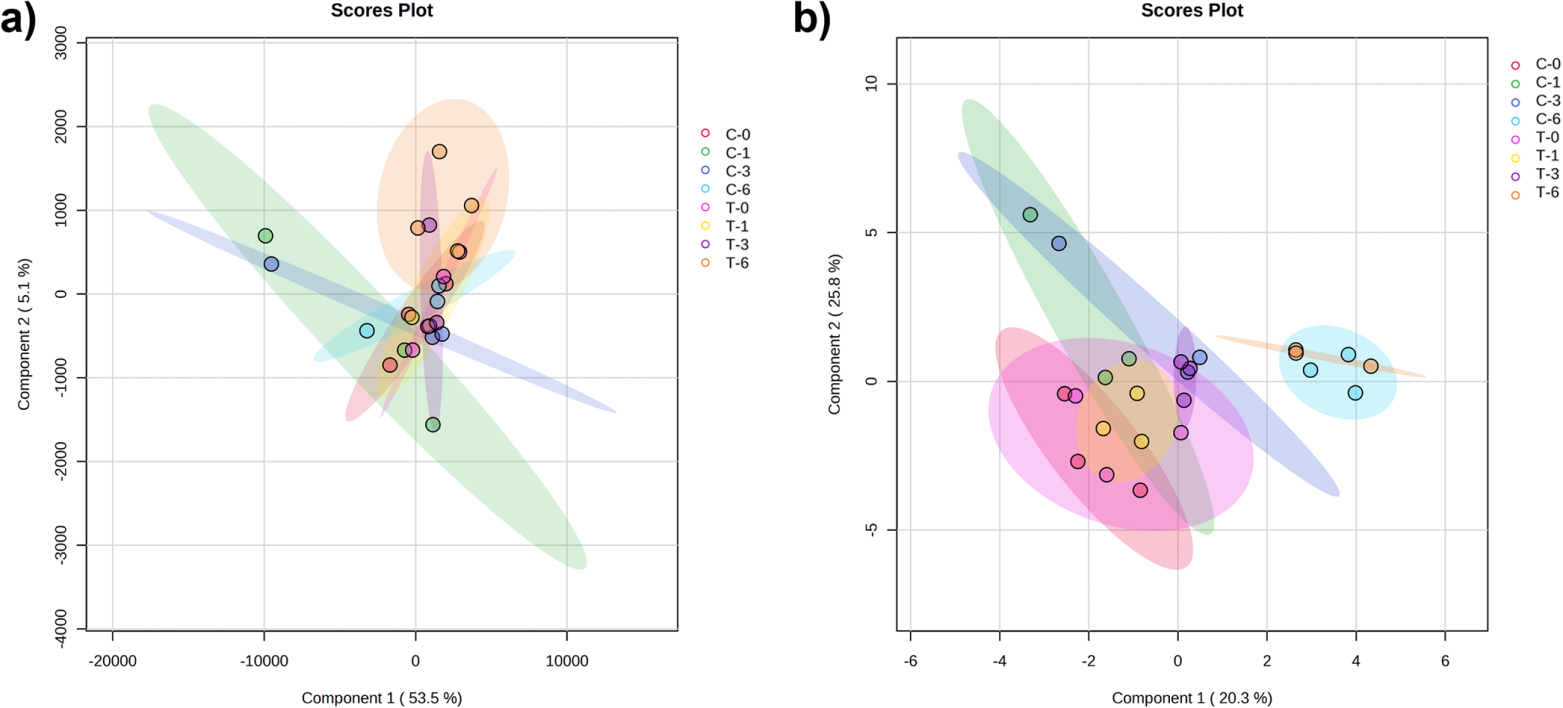
Analysis of the metabolomics data acquired upon sampling at different storage days of untreated (C) and plasma-treated (T) strawberry fruits with partial least-squares discriminant analysis (PLS-DA) and sparse partial least-squares discriminant analysis (sPLS-DA). (a) Score plots obtained by PLS-DA and (b) sPLS-DA.

Metabolic features significantly affected by the storage time were presented in Figures 3 and 4, and they were found to have a relatively high variable importance in projection (VIP) score in the sPLS-DA and were also determined by two-way ANOVA (*p* < 0.05) box plots.

**Figure 3:**
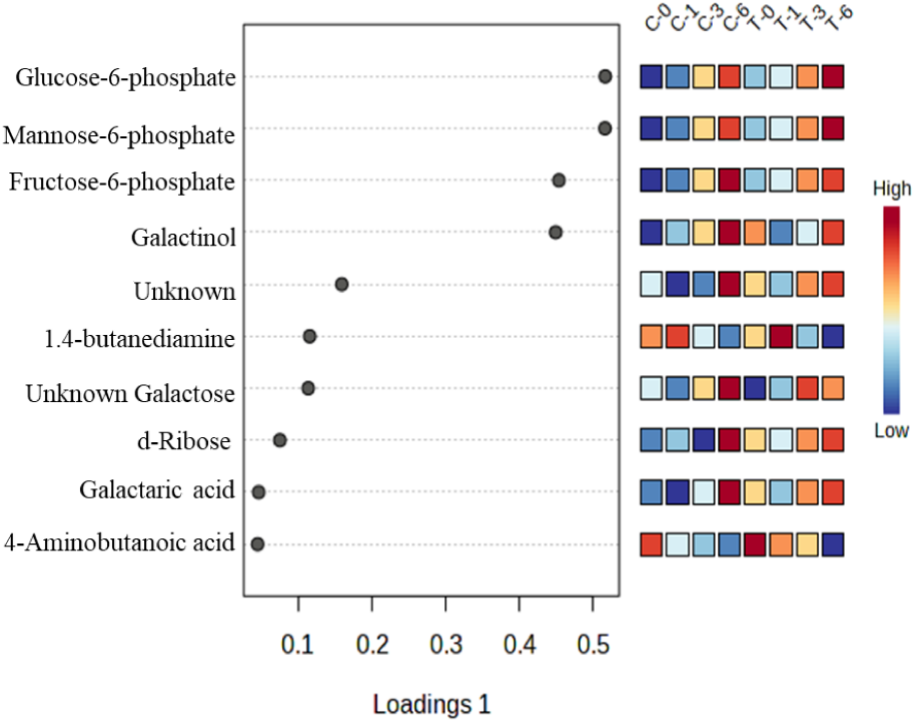
Top 10 metabolites with the highest variables in projection (VIP) scores of sPLS-DA (C).

**Figure 4:**
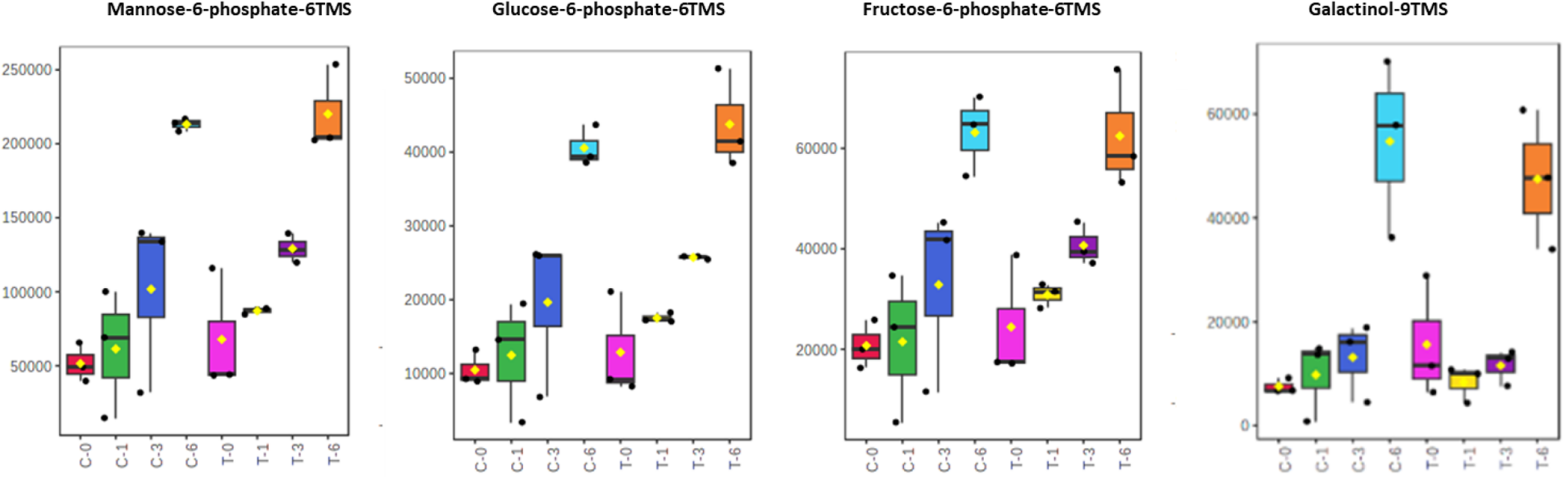
Box plots of different metabolites of strawberry samples obtained from both control (C) and plasma-treated (T) samples at different storage days at *p* < 0.05. The bar plots show the original values (mean ± SD).

Storage of fruits can have various effects on their chemical composition, including changes in the levels of sugars and other metabolites.^57^ In the present study, mannose, glucose phosphates, and other sugars increased during storage in both control and treated samples. The increase of sugars in strawberries can enhance their sweetness and flavor, making them more desirable to consumers. However, on the negative side, it can also lead to browning and softening of fruits, which can reduce their shelf life and overall quality. Studies indicate that reactive plasma species can break specific molecular bonds, leading to chemical modifications on the side chains of certain amino acids, particularly those with aromatic and sulfur residues, which are highly susceptible to these changes.^48,58^ For instance, direct exposure to argon plasma has been reported to degrade COOH and CNH_2_ groups in l-alanine.^59^ While most studies show that soluble sugar content often remains stable following plasma treatment, indicating minimal impact on major quality parameters.^60–62^ Some studies have reported significant decrease in sugar content, as in Chinese bayberries^63^ or increase, as observed in fresh-cut pears.^64^

Cold plasma treatment using the present parameters did not induce any significant (*p* > 0.05) changes in the strawberry primary metabolome up to day 6 of storage.

In conclusion, the study demonstrated that CP treatment effectively extended the shelf life of fresh-cut strawberries by 2–3 days by reducing loads of different spoilage microbial groups, suppressing yeast growth in the initial days of storage, and inhibiting lactic acid bacteria and Enterobacteriaceae. Notably, although microbial growth occurred mainly in the surviving mesophilic and psychrotrophic populations, plasma treatment delayed spoilage progression, suggesting its effectiveness as a microbial control measure. Furthermore, the plasma treatment did not adversely affect the strawberries’ physicochemical qualities, including TSS, hardness, and color, while maintaining their nutritional integrity. The treatment caused a slight reduction in ascorbic acid immediately, yet preserved its stability over the storage period. In terms of antioxidant properties, CP treatment enhanced the DPPH activity and maintained the total phenolic content (TPC) with minimal variation. From a metabolomics perspective, CP showed minimal impact on primary metabolites, such as sugars and acids, which are crucial for maintaining flavor and taste. Results indicate the treatment’s suitability for enhancing strawberry quality during storage.

It is important to highlight, however, that although cold plasma has shown promising results in the food sector, its applicability is still hindered by various factors, such as scalability issues and toxicological effects on food. Further research should therefore focus on clarifying these issues.

## Supplementary Material





## Notes

Figure S1: representative total ion chromatogram (TIC) of the control strawberry sample showing the major detected primary metabolites; Figure S2: primary metabolomic analyses of strawberry samples; Table S1: HPLC–MS/MS acquisition parameters (dynamic MRM mode) used for the analysis of the phenolic compounds; Table S2: gas chromatographic (GC) separation conditions and electron ionization-quadrupole-mass spectrometry (EI-Q-MS) settings for analysis of strawberries primary metabolites (PDF)
Table S3: twenty-seven unidentified metabolites were labeled as “Unknown,” with annotations including retention time (tR) and retention index (RI) (XLSX)
