# Supplementary material for: Impact of Cold Plasma Treatment on the Shelf Life and Metabolite Profiles of Strawberries during Storage
Source: ACS Food Sci Technol. 2025 Oct 3;5(10):3929–41. doi: 10.1021/acsfoodscitech.5c00700 (PMC12538710; doi:10.1021/acsfoodscitech.5c00700)
Supplement: Supplementary file 1 [file fs5c00700_si_002.pdf]

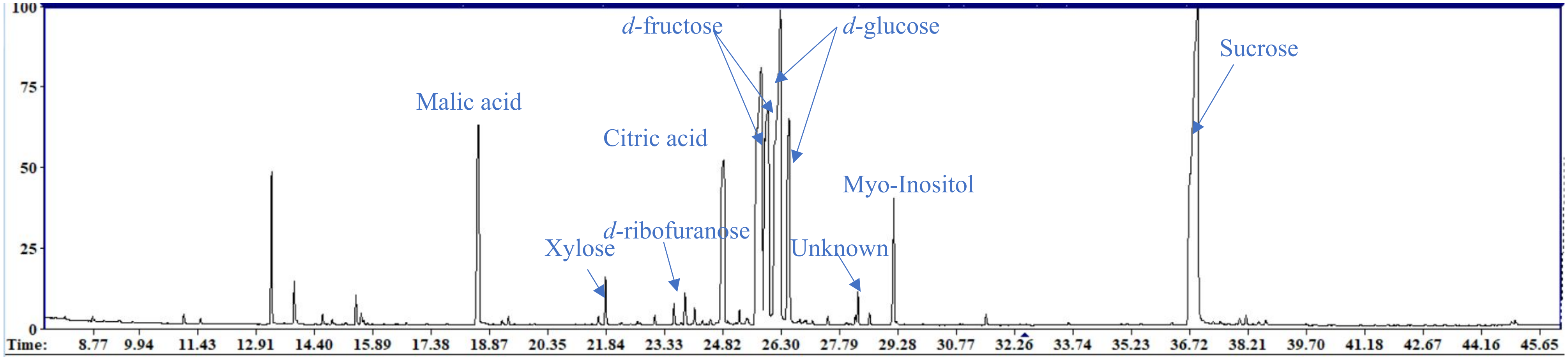

Figure S-1. Representative total ion chromatogram (TIC) of the control strawberry sample showing the major detected primary metabolites.

A

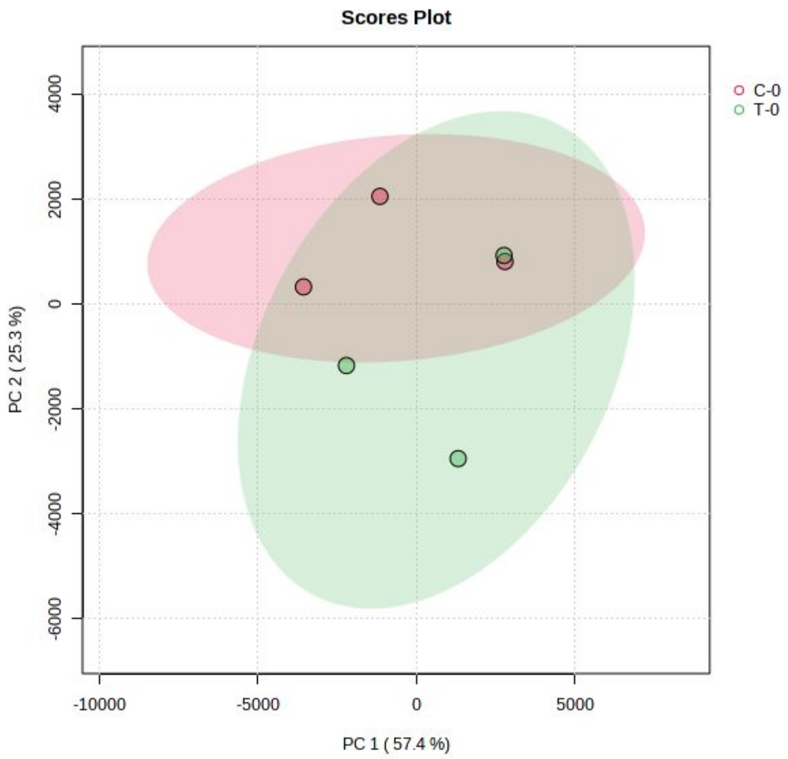

B

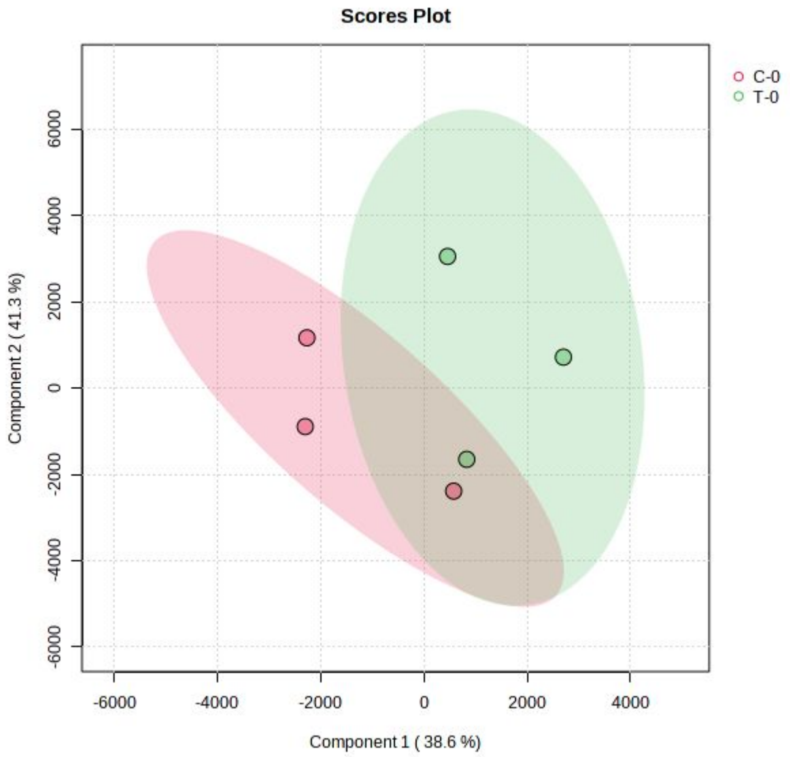

Figure S-2. Primary metabolomic analyses of both control (C-0) and DBD treated strawberries (T-0) at day 0. A) Scores plot of Principal component analysis (PCA); B)partial least square discriminant analysis (PLS-DA).

Table S1 HPLC–MS/MS acquisition parameters (dynamic MRM mode) used for the analysis of the phenolic compounds.

No.	Compounds	Precursor ion, *m/z*	Product ion, *m/z*	Polarity	Retention time (Rt, min)
1	Gallic acid	169	125.2	Negative	6.96
2	Neochlorogenic acid	353	191.2, 179	Negative	9.52
3	Delphindin-3-galactoside	465.01	303	Positive	11.36
4	(+)-Catechin	289	245.2,109.2,123.2	Negative	11.44
5	Procyanidin B2	576.99	576.99, 321.2	Negative	12.41
6	Chlorogenic acid	353	191.2, 127.5	Negative	12.42
7	*p*-Hydroxybenzoic acid	137	93.2	Negative	12.86
8	(-)-Epicatechin	289	245.1, 109.1, 123.1	Negative	13.03
9	Cyanidin-3-glucoside	449	287.3, 255.6	Positive	13.14
10	Petunidin-3-glucoside	479.01	317, 302, 186.2	Positive	13.26
11	3-Hydroxybenzoic acid	137	93.2	Negative	13.59
12	Caffeic acid	179	135.2, 134.1	Negative	13.65
13	Vanillic acid	167	152.4, 108.1	Negative	14.32
14	Pelargonidin-3-glucoside	433.01	271, 121	Positive	14.52
15	Pelagonidin-3-rutinoside	579.01	271	Positive	14.56
16	Malvidin-3-galactoside	493.01	331, 315.1, 287	Positive	14.64
17	Syringic acid	196.9	182.2, 121.2	Negative	15.28
18	Procyanidin A2	575	575, 285, 321.7	Negative	16.18
19	*p*-Coumaric acid	163	119.2, 93.2	Negative	16.70
20	Ferulic acid	193	134.2, 131.6	Negative	17.10
21	3,5-Dicaffeoylquinic acid	514.9	353.1, 191	Negative	17.61
22	Rutin	609	300.2, 271.2	Negative	17.73
23	Hyperoside	465.01	303, 61.1, 85	Positive	18.33
24	Isoquercitrin	463	271.2, 300.2	Negative	18.36
25	Delphindin-3,5-diglucoside	462.9	300.1	Negative	18.38
26	Phloridzin	435.39	273, 167, 123	Negative	18.83
27	Quercitrin	446.99	300.2, 301.2, 271.2	Negative	19.61
28	Myricetin	316.99	179.1, 182, 102	Negative	19.61
29	Naringin	578.99	271.3, 151.3	Negative	19.62
30	Kaempferol-3-glucoside	447	284.2,255.2, 227.3	Negative	19.77
31	Hesperidin	611.01	303, 334.8, 352.1	Positive	20.19
32	Ellagic acid	301	301, 229	Negative	21.41
33	Quercetin	300.99	151.2, 179.2, 107.2	Negative	21.87
34	Phloretin	272.99	167, 123, 81	Negative	22.30
35	Kaempferol	287.01	153, 69.1, 121	Positive	23.84
36	Isorhamnetin	314.99	300.2, 196.1	Negative	24.57

Table S2 Gas chromatographic (GC) separation conditions and electron ionization-quadrupole-mass spectrometry (EI-Q-MS) settings for analysis of strawberries primary metabolites.

Parameters	Setting
	**GC Settings**
**Separation column**	HP-5 capillary column (30 m × 0.25 mm ID, 0.25 µm film thickness, Thermo Fisher Scientific, Bremen, Germany)
**Carrier gas / carrier gas flow rate**	Helium / 1 mL/min
**Injector operation mode**	Splitless mode (90 s splitless time)
**Injector temperature**	250°C
**Temperature program**	1 min at 40°C, ramp 15°C/min to 70°C, 1 min at 70°C, ramp 6°C/min to 320°C, 10 min at 320°C
**MS Settings**	
**Ionization mode**	Electron ionization (EI)
**Electron energy**	70 eV
**Operation mode**	Scanning at 0.34 sec $scan^{-1}$
**m/z range**	50 - 500
**Resolution**	60,000
**Transfer line temperature**	250°C
**Ion source temperature**	250°C

The analysis was accomplished with GC2010 gas chromatography coupled online to a quadrupole mass selective detector Shimadzu GCMS QP2010, equipped with a CTC GC PAL liquid injector (Shimadzu Deutschland GmbH, Duisburg, Germany).
